# Supplementary material for: Child and Adult Care Food Program: Family Childcare Home Providers’ Perceptions of Impacts of Increased Meal and Snack Reimbursement Rates during the COVID-19 Pandemic
Source: Nutrients. 2024 Sep 25;16(19):3241. doi: 10.3390/nu16193241 (PMC11478229; doi:10.3390/nu16193241)
Supplement: Supplementary file 1 [file nutrients-16-03241-s001.zip › nutrients-3183585-supplementary.pdf]

nutrients MDPI

**Supplemental Table S1.** Perceived ease in implementing Child and Adult Care Food Program (CACFP) best practices for children by tier 1 (*n*=261) and tier 2 (*n*=257) family childcare home providers while receiving higher reimbursements.

| Ease of Implementing CACFP Best Practice | Tier 1 | Tier 2 |
|---|---|---|
| | *n* (%) | |
| **Whole fruits more often than fruit juice** | | |
| A lot harder | 19 (9.9) | 23 (12.6) |
| A little harder | 39 (20.3) | 35 (19.2) |
| A little easier | 64 (33.3) | 41 (22.5) |
| A lot easier | 70 (36.5) | 83 (45.6) |
| **Only natural, low-fat/reduced fat-cheeses** | | |
| A lot harder | 17 (8.8) | 21 (11.0) |
| A little harder | 50 (25.9) | 35 (18.3) |
| A little easier | 62 (32.1) | 53 (27.7) |
| A lot easier | 64 (33.2) | 82 (42.9) |
| **Dark green, red/orange, legumes, starchy, and other vegetables at least once per week** | | |
| A lot harder | 20 (9.7) | 19 (9.7) |
| A little harder | 47 (22.8) | 36 (18.5) |
| A little easier | 76 (36.9) | 60 (30.8) |
| A lot easier | 63 (30.6) | 80 (41.0) |
| **Whole grain-rich grains at least twice per day** | | |
| A lot harder | 17 (8.4) | 24 (12.1) |
| A little harder | 46 (22.7) | 40 (20.2) |
| A little easier | 80 (39.4) | 52 (26.3) |
| A lot easier | 60 (29.6) | 82 (41.4) |
| **Fruit and/or vegetable for snack** | | |
| A lot harder | 22 (10.8) | 28 (14.2) |
| A little harder | 53 (26.1) | 34 (17.3) |
| A little easier | 67 (33.0) | 49 (24.9) |
| A lot easier | 61 (30.0) | 86 (43.7) |
| **Processed meats no more than once per week** | | |
| A lot harder | 23 (13.1) | 17 (10.2) |
| A little harder | 44 (25.1) | 34 (20.5) |
| A little easier | 56 (32.0) | 44 (26.5) |
| A lot easier | 52 (29.7) | 71 (42.8) |
| **Only lean meats, nuts and legumes** | | |
| A lot harder | 21 (11.1) | 23 (12.2) |
| A little harder | 51 (27.0) | 35 (18.6) |
| A little easier | 71 (37.6) | 54 (28.7) |
| A lot easier | 46 (24.3) | 76 (40.4) |

nutrients

MDPI

**Supplemental Table S2.** Changes anticipated by tier 1 ($n$=261) and tier 2 ($n$=257) family childcare home providers when the tiered Child and Adult Care Program (CACFP) reimbursements are reinstated.

| Likelihood of anticipated change | Tier 1 | Tier 2 |
|---|---|---|
| | *n* (%) | |
| **Cost charged for childcare will increase** | | |
| Not likely | 79 (31.2) | 71 (29.1) |
| Somewhat likely | 109 (43.1) | 80 (32.8) |
| Extremely likely | 65 (25.7) | 93 (38.1) |
| **Variety of foods provided will decrease** | | |
| Not likely | 84 (33.1) | 63 (25.6) |
| Somewhat likely | 108 (42.5) | 109 (44.3) |
| Extremely likely | 62 (24.4) | 74 (30.1) |
| **Quality of foods provided will decrease** | | |
| Not likely | 114 (45.4) | 114 (46.5) |
| Somewhat likely | 87 (34.7) | 80 (32.7) |
| Extremely likely | 50 (19.9) | 51 (20.8) |
| **Meals/snacks provided will be less appealing to children** | | |
| Not likely | 118 (47.2) | 110 (44.7) |
| Somewhat likely | 87 (34.8) | 87 (35.4) |
| Extremely likely | 45 (18.0) | 49 (19.9) |
| **Number meals/snacks provided will decrease** | | |
| Not likely | 121 (47.8) | 131 (52.4) |
| Somewhat likely | 86 (34.0) | 63 (25.2) |
| Extremely likely | 46 (18.2) | 56 (22.4) |
| **Healthiness of foods provided will decrease** | | |
| Not likely | 129 (51.0) | 116 (47.2) |
| Somewhat likely | 79 (31.2) | 81 (32.9) |
| Extremely likely | 45 (17.8) | 49 (19.9) |
| **Will leave CACFP and parents/guardians will have to provide food** | | |
| Not likely | 159 (63.3) | 145 (59.4) |
| Somewhat likely | 58 (23.1) | 70 (28.7) |
| Extremely likely | 34 (13.5) | 29 (11.9) |
| **Will leave CACFP but continue to offer food** | | |
| Not likely | 177 (70.5) | 157 (63.8) |
| Somewhat likely | 48 (19.1) | 58 (23.6) |
| Extremely likely | 26 (10.4) | 31 (12.6) |
| **Will close business** | | |
| Not likely | 210 (83.7) | 206 (85.1) |
| Somewhat likely | 31 (12.4) | 29 (12.0) |
| Extremely likely | 10 (4.0) | 7 (2.9) |
